# Commentary to medical genetics and genomic medicine in Chile: Chilean experience on molecular diagnosis for neurodegenerative disorders

**DOI:** 10.1002/mgg3.288

**Published:** 2017-05-02

**Authors:** Marcelo Miranda, María Leonor Bustamante

**Affiliations:** ^1^ Department of Neurology Clínica Las Condes Av. Lo Fontecilla 441 Las Condes Santiago Chile; ^2^ Program of Human Genetics Biomedical Sciences Institute Av. Independencia 1027 Independencia Santiago Chile; ^3^ Department of Psychiatry and Mental Health North Division Faculty of Medicine University of Chile Av. Independencia 1027 Independencia Santiago Chile

## Abstract

In this article, the experience in the molecular diagnosis in neurodegenerative disorders in Chile, including present challenges and potential new pathways for development, is explained.

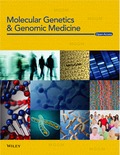

In her 2015 article from the series *Medical Genetics and Genomic Medicine Around the World*, Castillo‐Taucher refers to the scarcity of available resources for the molecular diagnosis of genetic conditions in Chile. As noted by Margarit et al. (2013), the needs of the Chilean population are unmet in terms of registry of cases of genetic disorders, availability of clinical geneticists, and awareness of health professionals of such conditions. Also, there are few laboratories that can perform molecular testing and there is a lack of dialog between professionals with different expertise to deliver the results to the affected families.

These authors mention that a significant proportion of the molecular diagnoses are made in a case to case fashion, and that the areas that are more developed are developmental disorders and congenital malformations, and cancer. We find it relevant to comment on the experience in another field, that of hereditary neurodegenerative disorders. Several cases have been published where diagnosis has been established. In most of them, the molecular testing and analysis have been performed abroad. This has been possible through international collaborations. Therefore, it depends on a high level of motivation from health professionals that have networks with foreign teams. Importantly, often times it relies on the ability of the families to pay for the shipment and analysis of samples.

Cases where causative mutations have been identified include hereditary dementias (Sinning et al. 2010), neuromuscular disease (Kleinsteuber et al. 2000; Castiglioni et al. 2015), parkinsonism (Perez‐Pastene et al. 2007; Behrens et al. 2010), chorea (Miranda et al. 2007), and spinocerebellar ataxia (Miranda 2015).

The cases reported in the literature only tell the successful studies that achieved a molecular diagnosis, and include conditions with a known genetic cause diagnosable with specific tests. The authors are aware of other cases that have been partially analyzed through standard tests but have not been able to establish a diagnosis. In some cases, the study has been incomplete because the families are unable to afford the cost of a more comprehensive set of tests. The fact that international partners have participated has lowered the costs of testing, but this type of association also limits the availability of tests to those that interest both parties.

Another potential pitfall in the study of neurodegenerative disorders is the significant clinical overlap between conditions and the genetic heterogeneity of each of them. Because of this, in several cases, single gene or gene panel testing is not the most cost‐effective tool. Furthermore, it is possible that the molecular tests currently available do not capture variants unique to the Chilean. The genetic constitution of the Chilean population is unique, with differences in the frequency of several ancestry informative markers with respect to European and other Hispanic populations. These issues can be overcome by next‐generation sequencing. Clinical exome sequencing is expected to become in the short term the first‐line test for selected cases of neurological disorders (Fogel et al. 2016). In Chile, the availability of next‐generation sequencing is growing. Currently, it is mainly based in academic settings for research purposes. Therefore, these resources do not solve the needs found in medical practice.

We believe that our local scientific community should foster the development of genomic capacities for improving the care of patients with neurologic conditions. Genetic neurodegenerative disorders account for a relatively small proportion of all cases, but for those affected, the identification of the cause can have a major effect. A critical factor that determines the benefit of a genetic diagnosis is the context in which is performed. Local interdisciplinary teams including all the specialists required to provide care for the patients and their families, and that are aware of cultural variables, would represent a leap in the quality of our health system.

Favorable circumstances are occurring currently that could mean a boost for establishing the discipline of neurogenetics in Chile. For instance, the Chilean government has launched this year a National Program for the Dementias. Among the actions promoted by the program is improving the systems for the diagnosis of these conditions. Furthermore, in our country there are well‐established groups that carry out research on cell biology of neurodegenerative disorders relevant to our population, such as Parkinson's disease, amyotrophic lateral sclerosis, and Creutzfeldt–Jakob disease. We hope that the work between the academic community, clinicians, and the public policy institutions will soon converge to fulfill another one of the needs of affected families.

## Conflict of Interest

None declared.
